# Study on Dynamic Mechanics of Node-Enhanced Graded Lattice Structure and Application Optimization in Automobile Energy Absorbing Box

**DOI:** 10.3390/ma16216893

**Published:** 2023-10-27

**Authors:** Bin Wu, Qiulong Chen, Fuyuan Liu, Min Chen, Yi Lu, Di Jiang, Yang Yi

**Affiliations:** 1 College of Mechanical and Electronic Engineering, Nanjing Forestry University, Nanjing 210037, China; 2 School of Advanced Technology, Xi’an Jiaotong-Liverpool University, Suzhou 215123, China

**Keywords:** graded lattice structure, dynamic mechanics, structure optimization, automobile energy absorbing box

## Abstract

Based on the lightweight characteristics of automotive energy absorption boxes and the requirement of good energy absorption effect, this article first applies the node-enhanced body centered cubic (NBCC) lattice structure to the inner core design of automotive energy absorption boxes. The gradient study of the NBCC lattice structure was carried out using a drop hammer impact and split Hopkinson pressure bar (SHPB). The results indicate that gradient lattice structures have advantages in energy absorption, but there are differences under different gradient strategies. When the impact is not sufficient to compact the structure, the vertical rod diameter gradient node-enhanced lattice structure (RGNBCC) can absorb more energy and improve energy absorption performance by 25%. The vertical height gradient node-enhanced lattice structure (HGNBCC) is more suitable for high-speed impact conditions. Based on the advantages of the RGNBCC in resisting low-speed impacts, it is applied to the inner core design of automotive energy absorption boxes and optimized using multi-objective optimization methods. The optimization results show that the maximum peak impact force is reduced by 45.6% and the specific energy absorption is increased by 30.4%.

## 1. Introduction

According to research findings, the probability of frontal collisions occurring in automobiles is as high as 66.9%. When a frontal collision occurs, the front bumper is initially subjected to the impact, after which the impact force is transmitted to the connected energy-absorbing box [1,2,3]. The energy-absorbing box absorbs a portion of the impact force through structural deformation, thereby reducing the damage caused by the collision to the vehicle and ensuring the safety of the occupants inside. Consequently, automotive energy-absorbing boxes enhance the passive safety performance of vehicles [4,5,6].

In recent years, lattice structures have garnered significant attention in engineering applications due to their low density and customizable mechanical properties. They provide broad application prospects for enhancing shock absorption and energy absorption [7,8,9,10,11]. Gradient lattice structures have become a common design concept known for their superior energy absorption capabilities compared to uniform lattice structures. Al-Saedi et al. [12] conducted finite element simulations and experimental tests to compare the mechanical behavior of rod-diameter gradient lattice structures under quasi-static compressive loading. The results revealed that the collapse process of gradient lattice structures occurred layer by layer, starting from the low-density layers and progressing towards the high-density layers. Such gradient lattice structures entered the initial stages of densification earlier than uniform lattice structures. However, gradient lattice structures were capable of absorbing higher amounts of energy. Bai et al. [13] investigated the static mechanical performance of gradient lattice structures with different unit cell sizes, emphasizing their supportive properties under small strains and outstanding energy absorption capabilities under large strains. This makes them suitable for impact protection devices. We previously added small spheres at the rod connection of the body-centered cubic (BCC) lattice structure for node strengthening design. Utilizing homogenization theory, we studied the elastic modulus of the lattice structure, and the results demonstrated that the equivalent elastic modulus of the node-enhanced NBCC lattice structure was 24.7% higher than that of the standard BCC lattice structure. Then, gradient design was carried out on the node-enhanced NBCC structure and quasi-static compression tests were conducted. The results revealed improvements in load-bearing capacity and energy absorption efficiency for the gradient node-enhanced BCC lattice structure [14]. However, these studies have been limited to static mechanical experiments, and the dynamic mechanical performance of lattice structures under various gradient strategies has yet to be further examined under dynamic impact conditions.

In order to maximize the energy absorption efficiency of energy-absorbing boxes, some scholars have employed lattice structures as the core of automotive energy absorbers and have conducted multi-objective optimization designs. Cetin et al. [15] introduced a body-centered cubic (BCC) lattice structure into the energy-absorbing box, demonstrating a significant increase in energy absorption under axial impacts. Zhou et al. [16] filled their energy-absorbing box with a negative Poisson’s ratio gradient lattice structure and investigated its crashworthiness and vibration resistance performance by comparing it with a uniform lattice structure filling. Subsequently, they optimized the thickness distribution of each layer of the lattice structure based on the NSGA-II algorithm. Leveraging the gradient characteristics of the human skeletal structure, Wang et al. [17] optimized the design of energy-absorbing boxes filled with negative Poisson’s ratio lattice structures using the NSGA-II algorithm. Furthermore, there have been multi-objective optimization designs based on gradient distributions resembling cacti [18] and honeycombs [19], which have enhanced the energy absorption effectiveness of automotive energy-absorbing boxes. However, most of the optimization designs for automotive energy-absorbing boxes have been achieved by adjusting the thickness of different layers of gradient lattice structures. Optimization designs for gradient rod diameter lattice structures in energy-absorbing boxes, especially in the form of nodal reinforcement, have not been widely explored in the current literature. Stylianos Kechagias et al. [20] conducted research on the connectivity of random lattice structures. Their study indicated that increasing the connectivity between rod diameters significantly improves the mechanical and fatigue performance of the structure. The rod diameter gradient design mode provides a smoother gradient distribution and does not disrupt the connectivity between the layers of the gradient lattice structure. Therefore, its application in the inner core design of automotive energy absorption boxes is worth further research.

This study is based on a node-enhanced lattice structure and further investigates the dynamic mechanical properties of lattice structures under different gradient strategy drop hammer impacts and SHPB testing. In addition, based on the lightweight characteristics and excellent energy absorption effect of lattice structures, the application of node-enhanced lattice structures in automotive energy absorption boxes has been explored. In order to analyze the load-bearing performance and energy absorption capacity of the lattice structure, we used a finite element simulation model based on the Johnson-Cook (JC) constitutive model. Based on the performance characteristics of a rod diameter gradient node-enhanced lattice structure (RGNBCC) under dynamic impact load, it is applied to the optimization of automotive energy absorption boxes. In order to fully utilize the advantages of gradient design and achieve better practical application results, we define structural design parameters as variables and adopt multi-objective optimization algorithms to obtain the best design results.

## 2. Materials and Methods

### 2.1. Structure Design and Material

**Figure 1:**
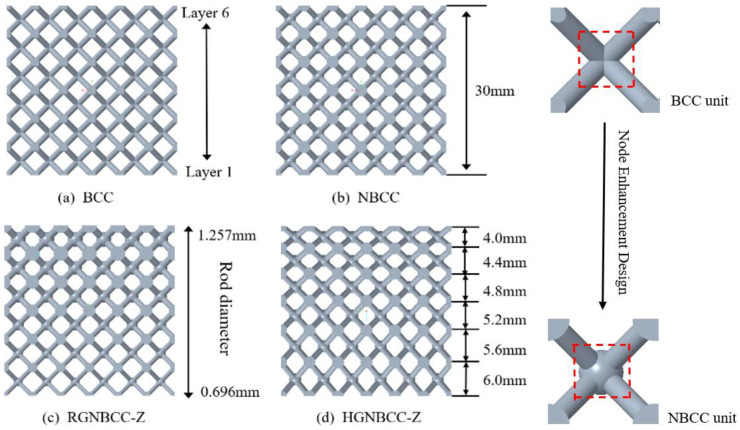
Schematic diagram of a node-enhanced union lattice structure and graded lattice structure (**a**) BCC; (**b**) node-enhanced lattice structure (NBCC); (**c**) vertical rod gradient NBCC (RGNBCC-Z); (**d**) vertical height gradient NBCC (HGNBCC-Z) [14]. (Get permissioned).

We design the lattice structure with the same volume. The rod type lattice structure is the most common type of lattice structure, among which a body-centered cubic (BCC) lattice structure, as shown in Figure 1a, is the most classic. It is mainly composed of four diagonally intersecting rods, with good load-bearing capacity and lightweight effects. However, there is a common problem of node stress concentration in rod type lattice structures, which greatly affects the load-bearing capacity [21]. In a previous study, on the basis of a BCC lattice structure design, our research group designed a node-enhanced lattice structure (NBCC) (see Figure 1b) to reduce the phenomenon of node stress concentration in the lattice structure, greatly ameliorating the problem of stress concentration and achieving satisfactory results. The corresponding graded lattice structure was designed based on two methods: rod diameter gradient and height gradient, which are shown in Figure 1c,d. 

It is important to note that although some soft materials exhibit good toughness and ductility, they often exhibit significant fracture due to their low strength during impact, making it difficult to ensure that the results under high-speed loads are not affected by instantaneous impact. Therefore, all the dynamic mechanical tests mentioned in this article used Ti-6Al-4V, which is a commonly used 3D printing material typically manufactured using Selective Laser Melting (SLM) technology and widely used in lattice structure research [22,23,24].

Different from quasi-static mechanical tests, the influence of strain rate on material properties cannot be neglected in dynamic mechanical tests. Ti-6Al-4V is a strain rate dependent material [24,25,26,27]. In the study of dynamic shocks, many researchers have chosen the Johnson-Cook (JC) model to describe constitutive relationships such as metals and rocks [28,29,30,31,32,33]. This model takes into account the strain hardening effect, and it covers both plastic and damage models of the material. The Johnson-Cook constitutive model describes plastic flow stress as [29]

(1) $$\sigma_{eq}=\left(A+B\varepsilon_{eq}^{n}\right)\left(1+Cln\frac{{\dot{\varepsilon }}_{eq}}{{\dot{\varepsilon }}_{0}}\right)\left(1-\frac{T-T_{r}}{T_{m}-T_{r}}\right)$$

where $\sigma_{eq}$ represents the von-Mises flow stress, *A* is initial yield stress, *B* reflects the hardening constant, *C* is the strain rate sensitivity factor, *n* denotes the hardening factor, and *m* is the temperature softening coefficient. ${\dot{\varepsilon }}_{eq},{\dot{\varepsilon }}_{0}$ represent the current strain rate and the reference strain rate. T, $T_{r}$, $T_{m}$ represent the working temperature, room temperature, and melting temperature, respectively.

The Johnson-Cook failure model is based on the value of the equivalent plastic strain at element integration points, which can be described as [30]

(2) $$\varepsilon_{D}=\left[D_{1}+D_{2}e^{\left(D_{3}\sigma^{*}\right)}\right]\left[1+D_{4}In\left({\dot{\varepsilon }}_{eq}^{*}\right)\right]\left[1+D_{5}{\left(T\right)}^{m}\right]$$

where $D_{1}$ to $D_{5}$ are material constants and $\sigma^{*}=p/\sigma_{eff}$ is the ratio of the mean stress to the von-Mises equivalent stress, called the stress triaxiality ratio. In this experiment, the temperature effect of the material was not considered. The density of Ti-6Al-4V was set to 4405 kg/m^3^, elastic modulus was 107 GPa, and Poisson’s ratio was 0.323. The Johnson-Cook model parameters employed in the current simulations were obtained from Refs. [32,33] and are listed in Table 1.

### 2.2. Finite Element Model

#### 2.2.1. Drop Hammer Impact Test

**Figure 2:**
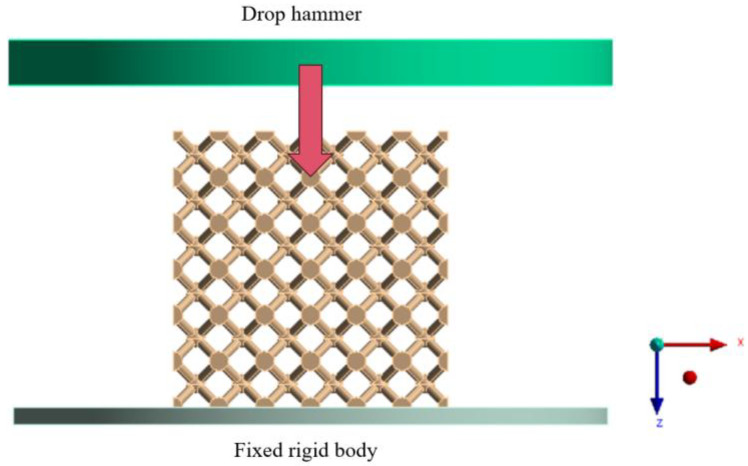
Schematic diagram of a finite element simulation of the drop hammer impact test.

The drop hammer impact simulation model is shown in Figure 2. The bottom of the sample structure was connected with the rigid body, and the top steel plate simulates the hammer. Two initial impact velocities were set, namely 7.5 m/s (${\dot{\varepsilon }}_{eq}=25\;s^{-1}$) and 19.5 m/s (${\dot{\varepsilon }}_{eq}=65\;s^{-1}$). The mass of the steel plate was 12 kg. Frictional contact with a friction factor of 0.1 was set in the tangential direction to simulate the contact condition between the flat plate and lattice structure, and the dynamic friction coefficient was set as 0.2. For the self-contact of lattice structures, we define a static friction coefficient of 0.1 and a dynamic friction coefficient of 0.2. Many scholars have introduced grid sensitivity analysis in lattice structure simulation research, which contributes to enhancing the accuracy and efficiency of simulations [34]. The choice of grid size is determined based on the unit quality and the computational time cost. After performing grid sensitivity analysis, the grid size for the upper and lower steel plates was set to 1 mm, while the grid size for the lattice structure was set to 0.2 mm. At this point, the average unit quality is 0.845, exceeding the 0.8 threshold, meeting the accuracy requirements for the simulation. We use a computer with a CPU of AMD Ryzen 7 5800H and a GPU of NVIDIA GeForce RTX 3060 (Manufacturer: Lenovo Co., Ltd., Beijing, China) for calculations. The calculation time for each model was approximately 12 h. The indicators under dynamic impact mainly include total energy absorption (EA), peak impact force (PCF), average impact force per length (MCF), and maximum displacement ($\delta_{max}$). These values can be derived from the analysis results, and PCF is the maximum value in the obtained force-displacement curve. MCF is the division of EA and ${\;\delta }_{max}$, which can be expressed as [35]

(3) $$MCF=\frac{\mathrm{EA}}{\delta_{max}}$$

#### 2.2.2. Split Hopkinson Pressure Bar (SHPB) Test

**Figure 3:**
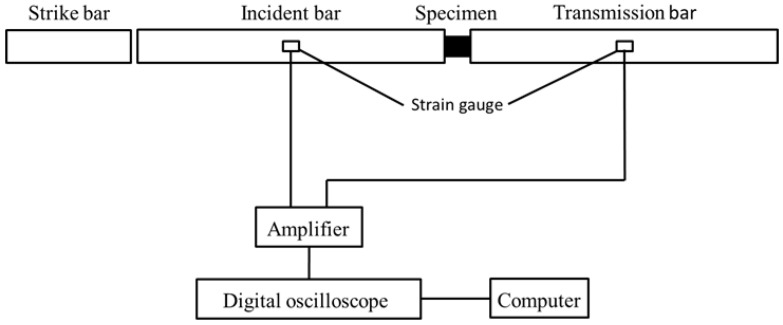
Schematic of an SHPB apparatus used for high strain rate impact loading.

The dynamic mechanical properties at high strain rate (10^2^–10^4^ s^−1^) can be accomplished using the split Hopkinson pressure bar (SHPB); more details about the SHPB can be found in Refs. [36,37,38]. Figure 3 is the illustration of the SHPB. The incident wave was $\varepsilon_{I}$, the reflected wave was $\varepsilon_{R}$, and the transmitted wave was $\varepsilon_{T}$. The dynamic mechanical properties of the tested specimens at a high strain rate can be obtained by calculation from Formulas (4)–(6) [37,38].

(4) $${\dot{\varepsilon }}_{s}\left(t\right)=-\frac{2C_{0}}{L}\varepsilon_{R}\left(t\right)=\frac{2C_{0}}{L}\left[\varepsilon_{I}\left(t\right)-\varepsilon_{T}\left(t\right)\right]$$

(5) $$\varepsilon_{s}\left(t\right)=-\frac{2C_{0}}{L}\int_{0}^{t}\varepsilon_{R}\left(t\right)dt=\frac{2C_{0}}{L}\int_{0}^{t}\left[\varepsilon_{I}\left(t\right)-\varepsilon_{T}\left(t\right)\right]dt$$

(6) $$\sigma_{s}\left(t\right)=\frac{E_{0}A}{A_{s}}\varepsilon_{T}\left(t\right)=\frac{E_{0}A}{A_{s}}\left[\varepsilon_{R}\left(t\right)+\varepsilon_{I}\left(t\right)\right]$$

where *L* denotes the length of the lattice structure, $C_{0}$ = $\sqrt{\frac{E_{0}}{\rho_{0}}}$ is the elastic wave speed of the pressure bars with $E_{0}$ and $\rho_{0}$ being the elastic modulus and density of the pressure bar material, and $A_{s}$ and *A* are the sectional areas of the sample and the bar, respectively.

**Figure 4:**
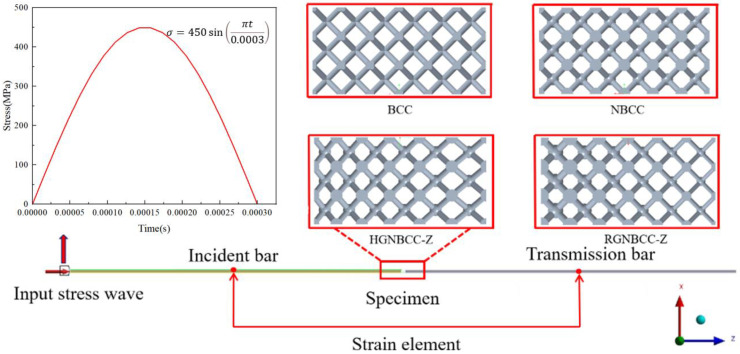
Schematic diagram of SHPB finite element analysis.

Previous studies have shown that bullet impacts form waves similar to half-sine waves at the end of the incident rod. Using a half-sine wave instead of bullet impact in finite element simulation can generate a smooth stress-strain curve [38]. The schematic diagram of the finite element analysis is shown in Figure 4. The software ANSYS 2022R1/LS-DYNA was used for SHPB simulation, and the hit bullet was replaced by a short-time half-sine wave. A nonreflective boundary condition was applied at the bottom of the transmission rod. In order to save calculation time, a 1/4 model was adopted and a symmetrical boundary condition was applied. The material of the incident rod and transmission rod was defined, with an elastic modulus of 70 GPa, Poisson’s ratio of 0.3, and density of 2730 kg/m^3^. The material of the lattice structure was Ti-6Al-4V. Through the element sensitivity analysis, the element size of the member was set as 3.0 mm, and the element size of the lattice structure was set as 0.3 mm. We used the same computer as above for calculation, and the calculation time for each SHPB model was approximately 4 h. In addition, the length of the rod must be able to avoid interference between the incident wave and the reflected wave. After calculation, the length of the incident rod and the transmission rod was set as 2600 mm, and the diameter was 50 mm. An element was extracted at the midpoint of the incident bar and the transmission bar, respectively, and its strain-time data was exported. The strain rate at this time and the stress-strain curve of the lattice structure can be obtained according to Formulas (4)–(6).

## 3. Dynamic Simulation Results and Discussion

### 3.1. Drop Hammer Impact

**Figure 5:**
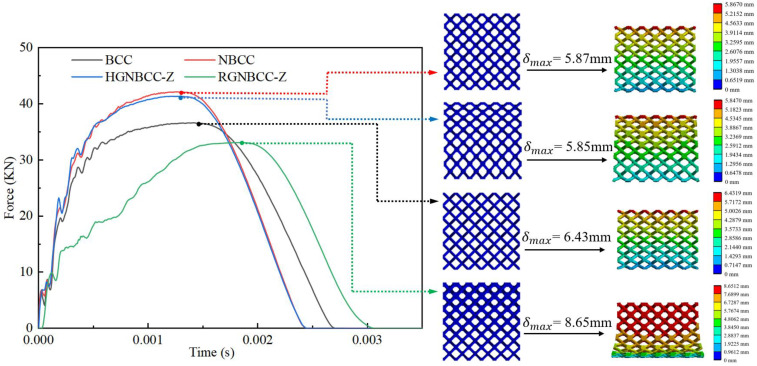
Force-time curve at impact velocity of 7.5 m/s.

The force-time curve at 7.5 m/s is shown in Figure 5. During the entire impact process, after the drop hammer contacts the lattice, its displacement initially increases, then decreases, and finally bounces back. The position of the point in the figure indicates that the lattice structure reaches its maximum impact compression displacement under impact conditions. It can be seen that the compression displacement of different lattice structures is diverse. The RGNBCC-Z obtains the maximum compression displacement due to the existence of its thin rod diameter. The compression displacement of the BCC is shorter, and the other two structures have the shortest compression displacements. The impact force shakes violently several times over time, gradually decreasing after reaching the maximum displacement point. When the falling hammer separates from the lattice, the impact force decreases to zero, and the impact process ends. During the entire impact process, the maximum peak impact force PCF of the NBCC is the highest, the HGNBCC-Z is slightly smaller, and the RGNBCC-Z has the minimum maximum peak impact force PCF. This was caused by its thin rod diameter layer, and its entire impact time was also the longest. 

**Figure 6:**
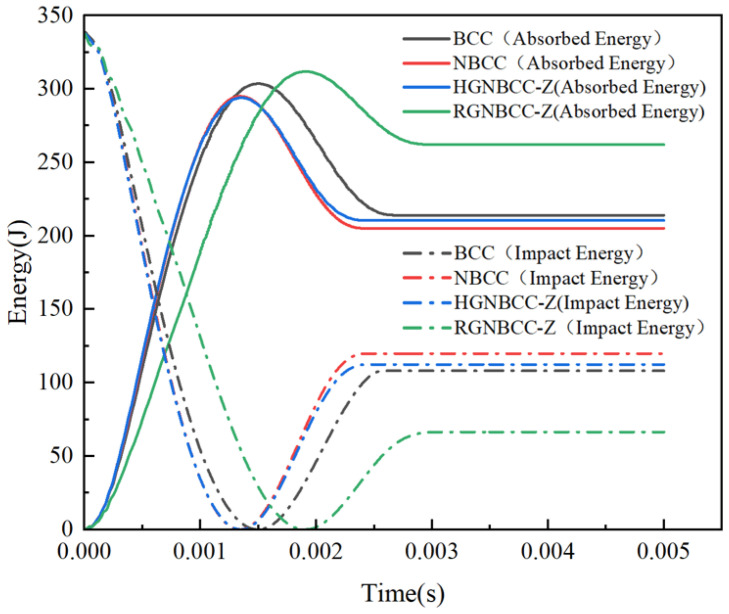
Energy-time curve at an impact velocity of 7.5 m/s.

Figure 6 shows the energy-time curve at an initial speed of 7.5 m/s. During the impact process, the kinetic energy of the hammer gradually transforms into the internal energy absorbed by the lattice. When the lattice structure reaches its maximum displacement under impact, the internal energy reaches its maximum. Due to the elasticity of the material, part of the elastic potential energy is converted into the kinetic energy that pushes the hammer back up. At this time, the internal energy is gradually decreasing, and the kinetic energy is starting to rise. Finally, the drop hammer and lattice structure become completely separated, and the entire impact process ends. During the entire impact process, kinetic energy loss is caused by factors such as friction, resulting in the kinetic energy of the falling hammer being greater than the energy absorbed by the lattice structure. This phenomenon is consistent with the energy loss in the experiment. At this point, the internal energy and kinetic energy gradually tend to stabilize. From the abscissa point of view, the internal energy of the RGNBCC-Z reaches the highest value later, followed by the BCC structure, which means that they take a relatively long time to withstand the impact process. From the perspective of energy, the final stable internal energy value of the RGNBCC-Z is about 25% higher than that of the other lattice structure, and the other three types are not much different. This is because the RGNBCC-Z produces more plastic deformation during the impact process, and the energy absorption mainly depends on the plastic deformation of the structure, so the gradient characteristic of this structure has the advantage of absorbing energy before reaching densified. This characteristic has great application value for energy absorption under low-speed impact.

**Figure 7:**
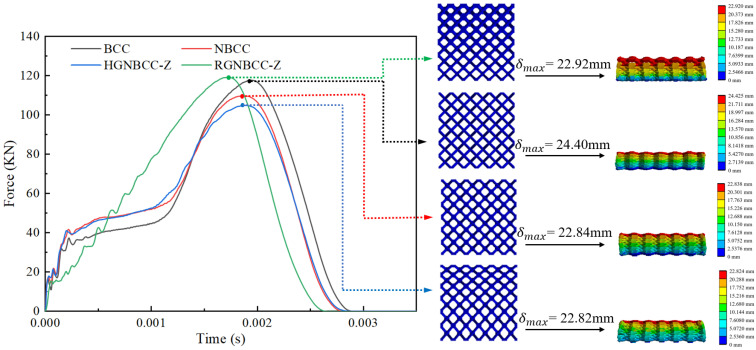
Force-time curve at an impact velocity of 19.5 m/s.

The force-time curve under the condition of an initial speed of 19.5 m/s is shown in Figure 7. Under the impact of this high speed, the lattice structure can reach densification, and the curve shows a different trend from that under the impact of 7.5 m/s speed. The highest point of the reaction force is the point with the largest impact displacement. The area before this point is similar to the quasi-static curve in trend. The RGNBCC-Z still shows an upward trend of wave jitter. During the initial period of impact, the reaction force of the BCC lattice structure is weaker than that of the NBCC and HGNBCC-Z. Before reaching its own densification point, the reaction force of the BCC lattice structure rises rapidly, surpassing the NBCC and HGNBCC-Z. This indicates that the BCC lattice structure exhibits a larger maximum peak force. After reaching the impact displacement limit, it starts to rebound, and the force gradually decreases and finally tends to zero. 

**Figure 8:**
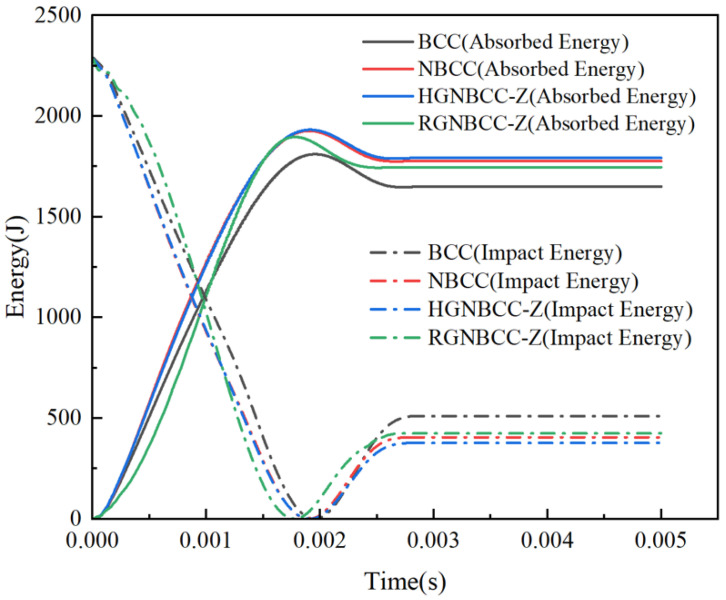
Energy-time curve at an impact velocity of 19.5 m/s.

Figure 8 shows the energy-time curve under high-speed impact. The global maximum energy absorption and final energy absorption of the BCC structure are both smaller than those of the node-enhanced lattice structure, reflecting the superior energy absorption effect of the node-enhanced lattice structure proposed in this paper under dynamic impact. The energy absorption of the RGNBCC-Z structure is lower than that of the NBCC and HGNBCC-Z structures, indicating that the advantage of the RGNBCC-Z thin rod diameter structure no longer exists when the crystal lattice is densified. In addition, from Figure 5 and Figure 7, it can be seen that the higher the impact velocity of the falling hammer, the greater the peak impact force on the lattice structure.

The parameters of the lattice structure in the impact process are listed in Table 2. It can be observed that the energy absorption efficiency increases with higher impact energy. Under low-speed impact, the MCF of the HGNBCC-Z is the highest, which demonstrates that the structure exhibits high energy absorption efficiency per unit displacement. Although the MCF of the RGNBCC-Z is the lowest, it exhibits the ability to absorb the most energy. Therefore, when the impact load is not sufficient to densify the lattice structure, this gradient design strategy can achieve a better energy absorption effect. Under high-speed impact, the node-enhanced lattice structure absorbs energy and the MCF better than the traditional BCC lattice, the HGNBCC-Z slightly better than the NBCC. Compared to the other two node-enhanced lattice structures, the RGNBCC-Z has no advantage in absorbing energy. This indicates that, under high-speed impact, all structures achieve densification, and the fine rod diameter of this structure cannot bring more plastic deformation than other structures. Therefore, under high-speed impact conditions that are sufficient to achieve densification of the lattice structure, choosing a height gradient to change the lattice structure will produce better results. The evaluation parameters of various impact load results under different gradient designs have their own advantages and disadvantages. In practical applications, appropriate lattice structures can be designed using different gradient methods according to specific requirements, thereby achieving better usage effects.

### 3.2. Split Hopkinson Pressure Bar (SHPB)

**Figure 9:**
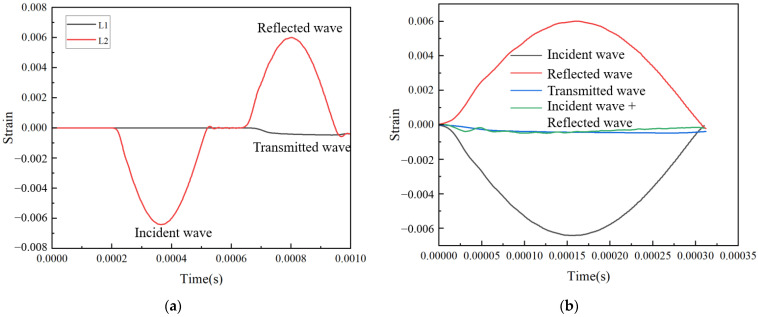
SHPB simulation data (**a**) BCC lattice structure waveform; (**b**) verification of homogenization assumption.

Figure 9a displays a group of typical waveforms of the BCC lattice structure in the SHPB test, where L1 represents the waveform from the transmission bar and L2 represents the waveform captured in the incident bar. The incident wave, reflected wave, and transmitted wave calculated from Figure 9a are illustrated in Figure 9b. To test whether the experimental assumption is satisfied in this case, the superimposed waveform of the incident and reflected waves is also plotted in Figure 9b. It can be observed from Figure 9b that the superposition waveform of the incident wave and the reflected wave has a high agreement with the wave shape of the transmitted wave, which satisfies the homogenization assumption during the SHPB process. At the same time, the strains of other elements in the same section were extracted. The results show that the waveforms extracted from the strain element in the same section coincide very well, which proves that the assumption of a one-dimensional stress wave is also met at this time, and the simulation results are reliable.

**Figure 10:**
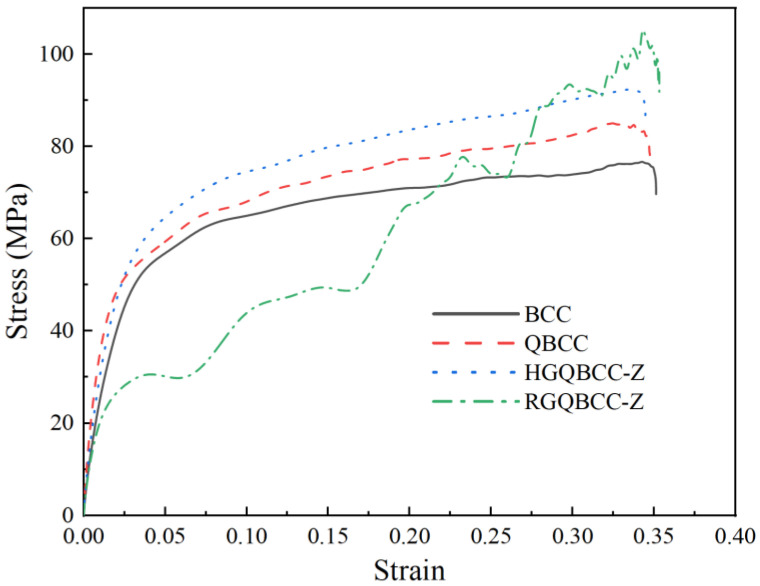
Stress-strain curve of a lattice structure at a high strain rate.

Based on Formulas (4)–(6), the stress-strain curve of the BCC, NBCC, HGNBCC-Z, and RGNBCC-Z at a high strain rate was obtained. After calculation, the strain rate at this time is 1843 s^−1^. It can be observed from Figure 10 that the vibration and jitter of the waveform are significantly reduced due to the application of half-sine wave, which enables a better representation of the mechanical properties of different lattice structures at high strain rates. The curves of the BCC, NBCC, and HGNBCC-Z mainly consist of linear elasticity and molding stages. Among them, the BCC exhibits the weakest bearing capacity under high strain rates, the HGNBCC-Z demonstrates the strongest mechanical properties, and the NBCC falls between these two types. The dynamic compression results of a lattice structure at a high strain rate are consistent with the trend of quasi-static compression, but the values are different. Both load bearing and energy absorption effects have been improved, which is consistent with the conclusions in another paper [37].

## 4. Optimization of an Automotive Energy Absorption Box Application Based on a Graded Lattice Structure

Through hammer impact testing of the lattice structure, it can be seen that the rod diameter graded lattice structure RGNBCC-Z has a higher energy absorption capacity in resisting low-speed impacts. In addition, the RGNBCC-Z lattice structure exhibits a lower peak impact force compared to other lattice structures in this paper. Therefore, based on the above two advantages, this structure is suitable for the inner core design of an automotive energy absorption box. This section starts with filling the automotive energy absorption box with a graded lattice structure of varying rod diameters, and multi-objective optimization design is carried out using graded lattice structure design parameters as optimization variables to obtain a better energy absorption box.

### 4.1. Parameter for Design of an Automotive Energy Absorption Box

**Figure 11:**
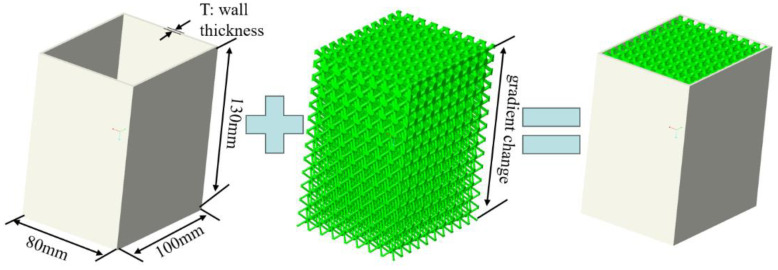
Composition of a lattice structure filled automotive energy absorption box.

The automobile energy absorption box is typically composed of thin-walled components that are formed through external stamping. It has an overall size of 80 mm × 100 mm × 130 mm and is made of aluminum alloy. The material parameters can be found in Table 3 [37]. An energy absorption box filled with a lattice structure is shown in Figure 11. The lightweight and high load-bearing capacity of the lattice structure can provide better performance for the energy absorption box. In order to study the collision performance of the energy absorption box, the impact test was conducted according to the requirements of the international RCRA standard for testing low-speed collisions of automobiles. The energy absorption box was subjected to an impact from an upper rigid plate weighing 0.8 t, at an initial speed of 15 km/h. The energy absorbing box adopts global automatic contact, with a static friction coefficient of 0.1 and dynamic friction coefficient of 0.2.

On the premise of ensuring that the total energy absorption meets the requirements, the design parameters of the automotive energy absorption box based on a gradient lattice structure are shown in Table 4. The graded lattice structure has a small rod diameter of *D*_1_, a large rod diameter of *D*_2_, a gradient change factor of M, and an energy absorbing box wall thickness of T. The important collision indicators of automotive energy absorbing boxes are peak contact force (PCF), specific energy absorption (SEA), and maximum collapse displacement of the energy absorption box ${\;\delta }_{max}$. The maximum peak impact force reflects the maximum force generated in a car collision. Excessive PCF can cause harm to the entire vehicle and passengers, so it is necessary to minimize this value as much as possible. The larger the specific energy absorption (SEA), the more energy absorbed by the unit mass energy absorption box. It can be expressed as [39]

(7) $$\mathrm{SEA}=\frac{ED}{m}$$

where *ED* represents the energy absorbed by the energy absorbing box during the entire collision process and m represents the mass of the energy absorbing box. $\delta_{max}$ represents the maximum travel distance. In order to protect the car, it should not exceed 60% of the length of the energy absorption box during a low-speed collision.

### 4.2. Multi-Objective Optimization Design Based on a Genetic Algorithm

#### 4.2.1. Design of Experiment (DOE) Method

Design of experiment (DOE) is an important sampling method, which provides sample points for optimizing the establishment of a surrogate model. The sampling methods mainly include central composite design (CCD), optimal space filling (OSF), and Box-Behnken and Latin hypercube sampling (LHS), among which Latin hypercube sampling was widely used because of its simplicity and effectiveness. To ensure high fitting accuracy of the established response surface, the sampling sample points should not be less than 2 n, where n refers to the number of parameters. In addition, the sampled samples should meet the design requirements of the automotive energy absorption box. To ensure that the total energy absorption meets the requirements, the parameter range of the sample is set (1 ≤ *D*_1_ ≤ 2, 2 ≤ *D*_2_ ≤ 3, 1 ≤ M ≤ 2, 2 ≤ T ≤ 2.4). Therefore, this article uses the Latin hypercube sampling method to randomly select 25 sample points within a numerical range and calculate and extract corresponding indicators. The design parameters and sample point results are shown in Table 5.

#### 4.2.2. The Response Surface Method and Sensitivity Analysis

**Figure 12:**
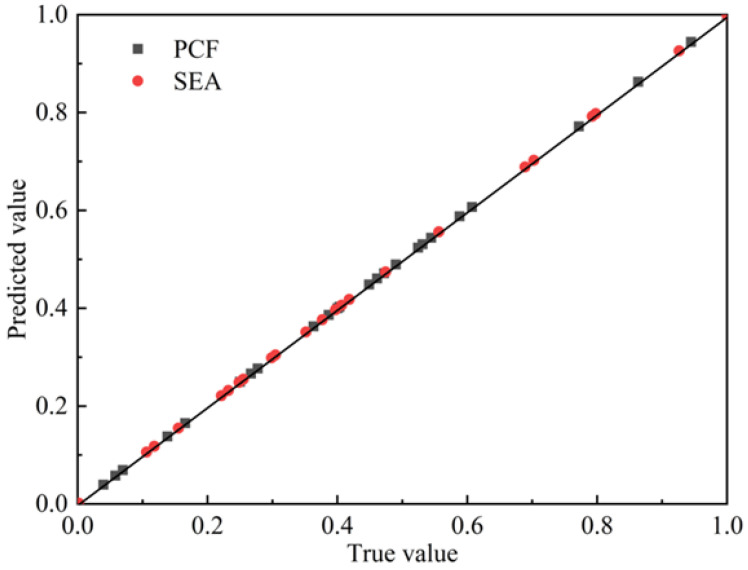
Scatter chart of predicted value and true value (normalized value).

Response Surface Methodology (RSM) is a method used to approximate the functional relationship between design variables, optimization objectives, and constraints through experimental design [40,41]. It mainly includes the polynomial response surface model, kriging model, radial basis function model, and artificial neural network model, etc. In this section, the kriging model is selected to fit the obtained sample points. We conducted response surface optimization using the dedicated module for response surface optimization in ANSYS Workbench. The result shows that the fitting coefficient R^2^ of PCF and SEA is 0.99, which can be used as the basis for the next optimization analysis. Figure 12 displays the fitting quality of the response surface. The *y*-axis represents the predicted values of the response surface, while the *x*-axis represents the true values of the design points. The scattered points align closely along the 45° line, indicating a strong agreement between the predicted and true values. The response surface fitting results are in line with expectations.

**Figure 13:**
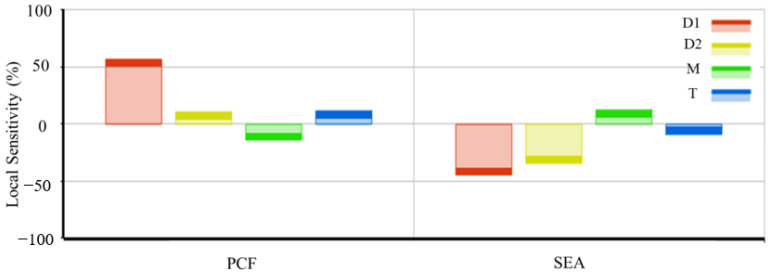
Sensitivity of design variables of an automobile energy absorption box to maximum peak contact force and specific energy absorption.

Sensitivity analysis can provide a more intuitive view for the degree of influence of each design variable on the output variable and references for overall optimization design. The sensitivity analysis of each design variable to the objective function is shown in Figure 13.

It can be observed that the diameter *D*_1_ of the large rod in the graded lattice structure has the most significant impact on the maximum peak impact force (PCF). Additionally, *D*_1_, *D*_2_, and T show a positive correlation, while the value of M exhibits a negative correlation. The diameter *D*_1_ of the large rod has the greatest impact on specific energy absorption (SEA), but it shows a negative correlation with it. *D*_2_ and T also show a negative correlation, while the M value shows a positive correlation. The influence of design parameters on PCF and SEA is inconsistent, so optimization algorithms need to be used to find the most suitable design that meets the constraint conditions.

From the above analysis, it can be seen that the small rod diameter *D*_1_, large rod diameter *D*_2_, gradient change factor M, and wall thickness T of the energy absorption box have a significant impact on the mechanical properties of the gradient lattice structure. In practical applications, it is necessary to consider the protection of passengers, so the maximum peak impact force of the energy absorption box should not be too large. Taking design factors into account, the optimization goal is set to be the maximum specific energy absorption, while ensuring that the maximum peak impact force is not more than 200 kN. Therefore, the multi-objective optimization design mathematical model of the automotive energy absorption box based on graded lattice structure is:

(8) $$\left\{\begin{array}{l} \;\mathrm{Min}\;\mathrm{PCF}\\ \;\mathrm{Max}\;\mathrm{SEA}\\ \;\mathrm{Subject}\;\mathrm{to}\;1\leq D_{1}\leq 2\\ \;2\leq D_{2}\leq 3\\ \;1\leq M\leq 2\\ \;2\leq T\leq 2.4\\ \;\mathrm{PCF}\leq 200\;\mathrm{kN} \end{array}\right.$$

### 4.3. Optimization Result and Discussion

The multi-objective genetic algorithm MOGA was used to solve and calculate the mathematical model. After iteration, the optimal design point was obtained, and its value was rounded to reconstruct the energy absorption box model, as shown in Table 6.

Multi-objective optimization is a powerful tool suitable for solving complex multi-objective problems. It provides comprehensive solutions to help decision-makers better understand problems, make wise decisions, and find the best balance between different objectives. However, in practical applications, it is also necessary to recognize the numerical error problem that exists in this method. Due to the random sampling within a certain range, there will inevitably be errors in sample selection. In addition, problems such as errors in the algorithm itself can also lead to imprecise results. Therefore, it is necessary to verify the optimization results. When verifying the optimization results, the boundary conditions, mesh size, and other settings of the finite element model were consistent with the previous text. As shown in Table 7, it can be seen that the error between the finite element verification results and the optimization prediction results provided by the response surface is relatively small, proving that the accuracy and reliability of the response surface optimization fitting meet the optimization requirements, and the results are reliable. Compared with the original uniform lattice filled energy absorption box, the optimized automotive energy absorption box with a graded lattice structure reduces the PCF by 45.6% and increases SEA by 30.4% compared to the energy absorption box. The effect of the optimization is significant, and the maximum compression distance $\delta_{max}$ meets the safety requirements at the same time, which improves the effectiveness of the car energy absorption box and reduces the damage to passengers caused by collision.

**Figure 14:**
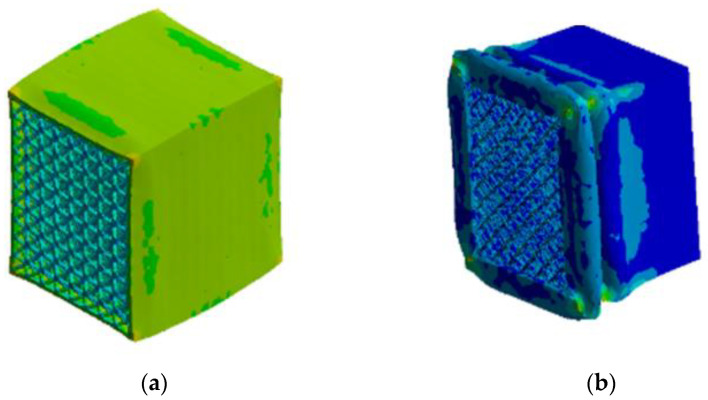
Comparison of deformation of automotive energy absorption box (**a**) before optimization, (**b**) after optimization.

**Figure 15:**
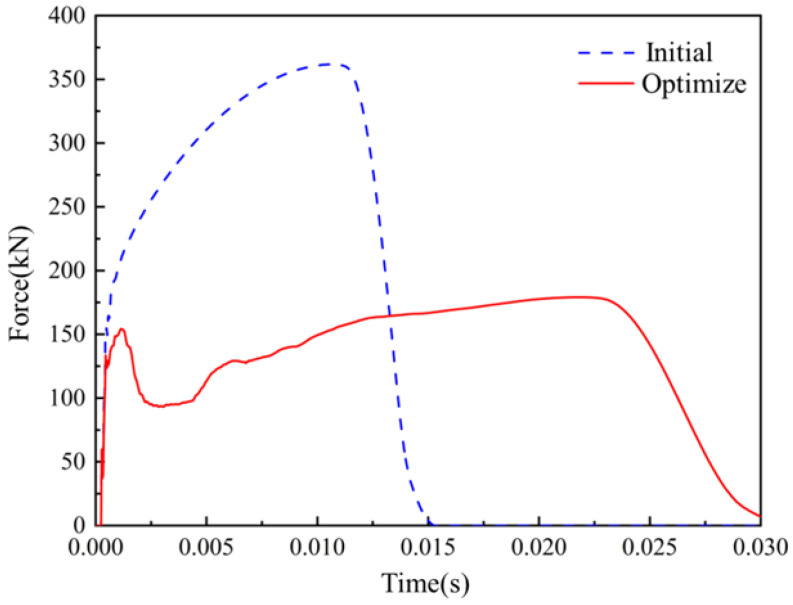
Force-time curve of an energy absorbing box during the impact process.

Figure 14 shows the comparison of the deformation of the automotive energy absorption box before and after optimization. After being impacted, the shell of the energy absorption box undergoes buckling deformation and expands outward. As the compression displacement continues to increase, the uneven stress distribution becomes more pronounced, resulting in local distortion and stress concentration. The energy absorption box filled with a uniform lattice before optimization starts to bulge in the middle, while the energy absorbing box based on graded lattice structure optimization starts to collapse and warp from the weak point of the rod diameter. Figure 15 shows the force-time curve of the energy absorbing box over time during the impact process. After the optimization, the maximum peak impact force and stress situation have significantly decreased, which means the optimized structure can better serve as the energy absorbing box. Essentially, the advantage of designing automotive energy absorption boxes based on graded lattice structure optimization lies in achieving higher energy absorption efficiency and lower peak contact force with more plastic deformation. This is a suitable optimization approach without exceeding the maximum stroke limit.

## 5. Conclusions

This article studies the dynamic mechanical properties of node-enhanced lattice structures and their graded lattice structure under medium and high strain rates and the optimization application of rod diameter gradient node-enhanced lattice structures (RGNBCCs) in automotive energy absorption boxes. The main conclusions are as follows:

(1) Under the impact load of a falling hammer, lattice structures with different impact energies will generate different compression deformations, and lattice structures designed with different gradient strategies will have different effects. When the impact is not enough to achieve structural densification, the gradient lattice structure RGNBCC-Z shows excellent energy absorption and impact resistance at low-speed impact. When the impact energy is large enough to achieve densification of the structure, the advantage of the rod diameter graded lattice structure RGNBCC-Z will no longer exist. In terms of peak impact force, the HGNBCC-Z is significantly smaller than the other structure, demonstrating good buffering performance under high-speed impact.
(2) Under high strain rate load impact, the load-bearing performance of a node-enhanced lattice structure (NBCC) is significantly enhanced compared to a BCC. The load-bearing capacity and energy absorption effect of the highly graded lattice structure HGNBCC-Z are greater than the NBCC. The RGNBCC-Z shows a wave like upward trend due to the presence of weak layers.
(3) Based on the graded lattice structure, the automotive energy absorption box was optimized. A multi-objective optimization algorithm was used to optimize the model. The optimization result shows that the maximum peak impact force was reduced by 45.6% and the specific energy absorption increased by 30.4%. The effect of the optimization was obvious, and it is of great practical significance to conduct structure optimization of automotive energy absorption boxes.

## Figures and Tables

**Table 1:** The material parameters in the Johnson-Cook model of Ti-6Al-4V.

*A* (MPa)	*B* (MPa)	*C*	*n*	*D*_1_	*D*_2_	*D*_3_	*D*_4_
1567	952	0.01	0.4	−0.09	0.25	−0.5	0.014

**Table 2:** Summary of a simulation lattice structure under dynamic impact.

Lattice Type	Impact Velocity (m/s)	Impact Energy (J)	$\delta_{max}\;$ (mm)	PCF (kN)	EA (J)	MCF (kN/m)
BCC	7.5	337.5	6.43	36.66	214.13	33.30
NBCC			5.87	42.13	205.16	34.95
HGNBCC-Z			5.85	41.34	210.56	35.99
RGNBCC-Z			8.65	33.12	262.14	30.31
BCC	19.5	2281.5	24.40	118.51	1650.2	67.63
NBCC			22.84	109.69	1776.9	77.80
HGNBCC-Z			22.82	104.88	1793.1	78.56
RGNBCC-Z			22.92	119.36	1746.6	76.20

**Table 3:** Aluminum alloy material parameters.

	Density (kg/m^3^)	Elastic Modulus (GPa)	Poisson’s Ratio	*A* (MPa)	*B* (MPa)	*C*	*n*
Value	2670	25.8	0.3	135	306	0.0069	0.619

**Table 4:** Design parameters value range.

Parameters	Description	Initial Value	Range
*D*_1_ (mm)	Small rod diameter	2	1–2
*D*_2_ (mm)	Large rod diameter	2	2–3
M	Gradient change factor	/	1–2
T (mm)	Box wall thickness	2.2	2–2.4

**Table 5:** Design parameters and sample point results.

Number	*D*_1_ (mm)	*D*_2_ (mm)	M (mm)	T (mm)	PCF (kN)	SEA (J/kg)
1	1.2	2.8	2	2.2	209,547	9213.314
2	1.5	2.42	1.06	2.184	298,457	8306.277
3	1.66	2.7	1.66	2.168	300,812	7776.364
4	1.58	2.94	1.54	2.152	312,349	7435.381
5	1.1	2.18	1.26	2.312	227,235	10260.39
6	1.94	2.62	1.22	2.344	417,333	6747.729
7	1.54	2.74	1.7	2.328	295,735	7878.949
8	1.9	2.26	1.58	2.2	387,136	7849.654
9	1.3	2.78	1.3	2.28	281,996	8071.654
10	1.22	2.22	1.98	2.392	233,254	10281.51
11	1.38	2.1	1.02	2.024	257,876	9861.545
12	1.18	2.9	1.46	2.216	255,562	8417.838
13	1.46	2.86	1.34	2.12	304,793	7729.13
14	1.7	2.38	1.86	2.04	285,008	8601.383
15	1.34	2.98	1.18	2.36	316,811	7218.179
16	1.82	2.34	1.74	2.136	313,861	8097.555
17	1.78	2.3	1.42	2.376	326,460	7877.582
18	1.62	2.54	1.9	2.056	285,672	8507.53
19	1.14	2.06	1.38	2.088	212,082	11180.59
20	1.06	2.66	1.94	2.104	205,468	10284.46
21	1.42	2.58	1.62	2.296	276,855	8549.234
22	1.86	2.82	1.1	2.008	367,005	6754.973
23	1.98	2.46	1.78	2.232	405,089	7270.836
24	1.26	2.14	1.14	2.264	251,850	9801.693
25	1.74	2.02	1.5	2.248	330,662	8850.027

**Table 6:** Response surface optimization design points.

Parameters	Initial Value	Optimization Value	Rounding Value
*D*_1_/mm	2	1.0398	1.04
*D*_2_/mm	2	2.6516	2.65
M/mm	/	1.8642	1.86
T/mm	2.2	2.0126	2.01

**Table 7:** Comparison between initial and optimization.

Object Variable	Initial Value	Optimization Value	Simulation Value
PCF (kN)	361.71	184.65	179.08
SEA (J/kg)	8329	10,728	10,396

## Data Availability

The data that support the findings of this study are available from the corresponding author, /Min Chen/, upon reasonable request.
